# Addressing Parental Smoking in Pediatric Settings of Chinese Hospitals: A Qualitative Study of Parents

**DOI:** 10.1155/2014/382345

**Published:** 2014-05-29

**Authors:** Abu S. Abdullah, Zhenyu Ma, Jing Liao, Kaiyong Huang, Li Yang, Zhiyong Zhang, Jonathan P. Winickoff, Guang-Min Nong

**Affiliations:** ^1^School of Public Health, Guangxi Medical University, Nanning, Guangxi 530021, China; ^2^Boston Medical Center, Boston University School of Medicine, Boston, MA 02118, USA; ^3^Department of Pediatrics, The First Affiliated Hospital of Guangxi Medical University, Nanning, Guangxi 530021, China; ^4^MGH Center for Child and Adolescent Health Research and Policy, Harvard Medical School, Boston, MA 02114, USA

## Abstract

This study explored factors associated with SHS exposure from parental smoking in Chinese families and assessed nature of antismoking discussions parents had with their children's pediatricians and how pediatricians might best engage with parents in an effort to reduce children's exposure to SHS. Six focus group discussions (FGDs) were conducted among 33 Chinese parents attending six major hospitals in Guangxi province, China. Most participants (32/33) had family members who smoke, and only 21% had strict restriction on smoking at home. Some parents did not know about health consequences of smoking and effects of SHS exposure on children. Situations that made it especially hard to avoid the child's SHS exposure were having an elderly smoker at home and having a visitor who smoked. Only few parents were asked by pediatricians about child's exposure to SHS at home, but only when child's illness was related to smoking. Parents believed that suggestions coming from pediatricians about smoke-free home and parental quitting would be acceptable to parents and other household members. The findings provide insight into SHS exposure reduction effort among Chinese parents and underscore the demand for pediatrician's engagement in addressing parental tobacco use.

## 1. Introduction

Tobacco use continues to be the leading global cause of preventable death. It kills approximately 6 million people each year, including more than 600,000 nonsmokers who die from exposure to tobacco smoke [1], of which 31% are children [2]. Since adopting the World Health Organization (WHO) Framework Convention on Tobacco Control (FCTC), more than 60 countries have initiated campaigns for smoke-free laws and over 17 countries now have a national law requiring all workplaces and public places to be smoke-free [3]. However, smoke-free laws do not protect children in the home or car, places where children spend much of their time. Although, China has initiated many tobacco control initiatives during the last decade, the prevalence of nonsmokers' exposure to second-hand smoke (SHS) or tobacco smoke pollution (TSP) [4] has remained relatively constant. National surveys on smoking behavior in 1996 and 2002 reported SHS exposure rate at 53% and 52%, respectively [5]. Another cross-sectional survey conducted in 2004 in six counties of China showed that 48.3% of the nonsmokers were exposed often or sometimes to SHS in their household [6]. Given the high prevalence of adult smoking in China, 52.9% in men and 2.4% in women [7], parental smoking has become the major cause of children's exposure to SHS. Studies have shown that the most common place where children are exposed to SHS is their own homes [8–10]. The harmful effects of child SHS exposure are well documented [11]. Several interventions have been implemented and evaluated [8, 12]. The pediatric setting can play an important role in encouraging parents to take measures that will protect their child from SHS exposure [13]. In China, almost 100% of all parents visit pediatricians in relation to the well child visits or other health-related issues during the early age of their child. Developing interventions that could be delivered through the pediatric setting would have great potential to reach a large number of parents who smoke.

We conducted this study to explore factors associated with SHS exposure from parental smoking in Chinese families and assess nature of antismoking discussions parents had with their child's pediatricians and how pediatricians might best engage with parents in an effort to reduce children's exposure to SHS. We conducted focus group discussions (FGDs) with parents of pediatric patients attending four Chinese hospitals. This study aims to provide a scientific basis for designing effective interventions to reduce children's SHS exposure in China.

## 2. Methods

### 2.1. Sample and Settings

Participants were parents of pediatric patients who were attending the departments of pediatrics in the selected six hospitals in four major cities of Guangxi province (a Southern Chinese province bordering Vietnam), China: First Affiliated Hospital of Guangxi Medical University (Nanning), Maternal and Child Health Hospital (Nanning), Liuzhou Maternal and Child Health Center (Liuzhou), Affiliated Hospital of Guilin Medical University (Guilin), Qinzhou Maternal and Child Health Center (Qinzhou), and Zhuxi Community Health Center (Nanning).

### 2.2. Procedures

Participants were recruited, during April-May 2013, through the hospital liaisons in each hospital who participated in the protocol development workshop of the project in an earlier stage. The liaison person, a senior pediatrician, was provided with the verbal and written background information of the study and the characteristics of people we were looking for to participate in the FGDs. Selection criteria were as follows: father or mother of a pediatric patient, smoker or nonsmoker or former smoker, willing to give consent to participate in FGD, and being able to communicate in Mandarin Chinese or local dialect (Cantonese). The hospital liaison person identified potential subjects and scheduled FGDs. We conveniently invited 5-6 interested parents to attend the FGD on a scheduled time slot.

### 2.3. Data Collection

A semistructured FGD guide was developed with reference to the research team's earlier work [14] and pilot tested with four parents resulting in minor changes. All of the FGDs were conducted in Mandarin Chinese and audio-recorded. The guide included questions and queries on the following themes: risks of smoking and SHS exposure, attitudes towards SHS, situations where children are exposed to SHS, measures taken to reduce child's exposure to SHS (if any), barriers encounters or potential barriers to reduce child's SHS exposure, experiences with the child's pediatrician about smoking or SHS exposure, and views about pediatrician engagement in promoting SHS exposure reduction and parental smoking cessation. Four interviewers conducted all the FGDs. Interviewers were graduate students at the School of Public Health of Guangxi Medical University and attended a 2-day training course on qualitative research methods and tobacco use reduction research. The training also included a session on the ethical aspects of human subject research. To collect data, two interviewers worked as a team; one moderated the FGD and the other took detailed notes and recorded the session with a digital voice recorder (with permission from the participants). All FGDs were held at the hospital in a private meeting room and lasted for approximately 90 minutes. The sessions started with the moderator explaining the purpose of the group discussion and assuring confidentiality of the data collected for the research project. To compensate for their time, each participant was given a cash amount of RMB 50 (US$8).

Written informed consent was obtained from each participating parent. The study was approved by the Ethics Committee of the Guangxi Medical University.

### 2.4. Analyses

The interviewers discussed and summarized the content of each FGD and reviewed the notes taken immediately after the FGD. These debriefings were useful (i) to identify most important themes and ideas and (ii) to assess the need for any modification in the subsequent FGD. The audio recordings were reviewed and transcribed for each group. Two members of the research team coded each transcript independently, with discrepancies resolved through consensus. The process of coding involved identifying key themes and marking these out on the transcripts [15]. All additional notes taken during the course of the focus groups were examined to identify various themes presented in these qualitative discussions.

## 3. Results

Six FGDs were conducted among 33 parents of pediatric patients from 6 hospitals in four major cities of Guangxi province, China. Twenty-two (66.7%) of the participants were males and 11 (33.3%) were females. Education varied from middle school or below (69.7%) to high school or above (30.3%). Close to half (45.5%) were nonsmoker, and 54.5% smoked (Tables 1 and 2).

The findings revealed six main themes relating to children's SHS exposure and parental smoking: attitude towards smoking in front of the child; attitude towards smoking in the car; attitude towards smoking and quitting smoking; knowledge of smoking and SHS; measures taken to reduce children's exposure to SHS; experience with and views about pediatricians inquiry about smoking and children's SHS exposure. These themes are described below supplemented by participants' statements on key themes provided in Table 3.

### 3.1. Attitude towards Smoking in front of the Child

#### 3.1.1. Household Members Smoking

The participants had varying views about household members smoking. Most (32/33) had family members in the household who smoke and family members interact frequently or occasionally. Some had strict restriction on smoking at home (21%, 6 male smoker and 1 female nonsmoker) while others would not dare to have smoking restrictions at home. Eleven parents said older family members (grandfather, grandmother, etc.) smoked at home and argued that, given the Chinese family structure where three generations of family live together, this would be impolite to ask elderly members not to smoke at home.

For example, one female nonsmoker whose daughter got Broncho pneumonitis said, “*I never smoke, my husband only smokes occasionally. But my father-in-law smokes a lot at home. He even smokes when he is cooking. His clothes and mouth always smell like smoke. When he hugs my daughter after smoking, I feel so bad but I cannot say anything*.”

Another smoking man said, “*generations of my family including grandfather, father, uncle, and I, are engaged in smoking at home; older members are accustomed to it, we have to respect their habits. While knowing that smoking in front of kids is bad, but there is no alternative other than smoking when I feel craving for cigarette*.”

#### 3.1.2. Friends Visit and Smoking

Entertaining friends with cigarettes when they visit home was common for both smokers (8/18) and nonsmokers (3/15). Some thought it would be embarrassing to ask a smoking guest not to smoke. A smoking father said, “*when friends came to drink at my home, all teased my son with a cigarette, I wanted my kid stay out, but he saw adults sitting there smoking, he was also eager to give it a try and I allowed*….*”* A nonsmoking mother said, “*Every time when guests are in the house, everywhere is smoky, I can only let kids go out and play*…*, but it is not possible always as I need to go out with him too.”*

#### 3.1.3. Smoking in Public Places

In situations where others were smoking in front of the children in public places, the majority (27/33, 82%) of participants felt that it was difficult to ask other people stop smoking, even if there was a smoke-free sign in place.

### 3.2. Attitude towards Smoking in the Car

Although all participants did not have a car, the views of smoking in the car (own car or taxi) were mixed. Several nonsmokers said that they hated cigarette smoke and when people were smoking in the car it makes them uncomfortable as they have to inhale fumes. A nonsmoking man said, “*when I went out with friends, if friends smoke, I would tell them to open the window,… only this choice seems to me reasonable. If I asked them to not to smoke in my car, instead, they laughed at me for being so greedy for owing a car!”*

Few smokers thought that smoking in the car is not a serious problem if they open the window, even when kids are there. Some parents expressed concern about bad smell in the car after smoking but were unable to elaborate much about the health hazards associated with these bed smelling chemicals.

### 3.3. Attitude towards Smoking and Quitting Smoking

Several smokers (7/18) described quitting smoking as a difficult task. A smoking man said, “*I also want to quit, but it is not easy to cut out the habitual craving on my own willpower. I smoke every day just like I take meal every day,… frankly speaking, I can give up a meal, but not the cigarettes*….”

Several smokers (6/18) had tried to quit smoking, but found it difficult to quit. A male smoker said, “*I tried to quit smoking many times, but I'm addicted. Several smoking parents thought smoking shows warm feeling and politeness in social engagements*. Few thought they are addicted and they like the smell of cigarettes.”

“*We gave up smoking during medical treatment, but after treatment we could not help it”* (a smoker who had pneumonia and another who had tuberculosis). Several nonsmokers (7 female and 2 male) had tried to persuade their family members to quit smoking, but to no avail. A father said, “*I am not smoking at all. My older brother and younger brother are both smokers. They smoke a lot. I told them about the hazards of smoking in the past thirty years, but they never listened to me.*”

### 3.4. Knowledge of Smoking and SHS

Some parents (10/33) were unable to correctly answer the questions about the health consequences of smoking and SHS exposure of children. Few parents thought their smoking caused no harm to the health of their child. Several parents said that they knew smoking and SHS were harmful to health, but they did not know any specific harms or how dangerous it could be. As one smoker father said, “*Hard to say, some people who never smoke also get lung cancer.”*

### 3.5. Measures Taken to Reduce Children's Exposure to SHS

Most parents had taken some sort of protective measures to prevent their child from SHS exposure. All female nonsmokers reported that they had taken “passive measures” against their children's exposure to SHS, such as taking kids away from the smoking places (home and public places) or opening the window at home or in the car. Some said applying or adopting measures to protect the child from SHS exposure is sometimes difficult in China when many people smoke.

“*In one occasion, I was on a bus with my daughter… a man sitting next to us started to smoke a cigarette. I told him that he should not smoke cigarettes in the bus. But he glanced at me and pretended not hearing me and continued smoking. I felt so angry and was not sure what to do*” (a nonsmoker mom).

The majority of smoking males (12/18) would smoke in the bathroom or balcony to reduce children's SHS exposure. Few said that they would do this in their own accord, while few would do this by coercion of nonsmoking family members. Some thought it would be fine to put the doors and windows open when people were smoking at home and, if possible, buy an air purifier (cleaner) to refresh the air.

### 3.6. Experience with and Views about Pediatrician Inquiry about Parental Smoking and Children's SHS Exposure

Few respondents (8/33, 24%) had positive experiences about the way they have been asked about SHS exposure of the children or about parental smoking status. Five of these respondents expressed that children's hospitalization due to respiratory diseases prompted doctor to ask them about their smoking behavior and practices at home. However, pediatricians did not enquire about smoking if the child's visit or hospitalization was related to nonrespiratory diseases. For example, a mom said: “*I visited pediatricians several times during the last year for fever and diarrhea of my baby, the doctor never asked me about smoking information.”*

A male smoker said, “*pediatricians never asked me about smoking during the few times I visited them last year. If smoking is really harmful to the child, they should ask and take action. *…* I hope doctors could ask and explain more about the pros and cons of smoking and tell us where to get medications for quitting.*”

“*I hope pediatricians would explain more about health hazards of smoking and SHS *…* and provide guidance about how we can make the home smoke-free. Doctors could give us antismoking stickers or posters to post at home *…* so others will see them and may not smoke*” (a female nonsmoker).

Most parents thought that advice from the pediatrician about the child's SHS exposure reduction or parental smoking cessation would be acceptable to Chinese parents.* “In Chinese society people respect doctors. If a message comes from the doctor, people will believe this and try to obey *…* if the doctor gives written information; we could show this to the smoking members of the family*” (a nonsmoker mom).

## 4. Discussion

Exposure to parental or household smoking was common among this sample of parents with children attending the pediatric departments in China. The high frequency of smoking at home when guests visited, which was reported by almost all parents in our study, is consistent with the findings of another local study [16]. During the last five years, China has launched a variety of tobacco control projects with support from international funding agencies. The Chinese government also enacted an indoor smoking ban in public places on May 1, 2011. Although smoking in public places may have decreased significantly, the prevalence of exposure to SHS at home among nonsmokers, including children, (67.33% in Jiangxi, 71.91% in Henan) is still very high [16]. From our findings, it is obvious that current Chinese cultural factors, which approve smoking, may play key roles in tobacco control and SHS exposure reduction in China. Why do parents of children feel that they have to expose their child to SHS from others? Possible reasons could be traditional Chinese cultural values which are pervasive in Chinese society and smoking culture, such as “to show respect to others,” “to be polite or friendly,” “to maintain the good relationships,” and “to develop business relationships” [17, 18]. For example, if parents tried to ask grandparents to stop smoking at home, it might be regarded as an affront or offensive to the elder. Also, sharing cigarettes with visiting guests is a way to welcome and establish rapport with guests [17, 19]. These kinds of social pressures represent the prevailing social norms surrounding smoking and could send mixed signals to smokers about the need to quit smoking. It is easy to see how the prevailing social pressures in China promote smoking among nonsmokers and work to maintain tobacco addiction among smokers. Physicians who highlight the ill effects of SHS exposure of children may help to shift social pressures toward healthier behavioral norms.

The findings show that some parents had incorrect knowledge about the hazards of smoking and SHS exposure of children. Earlier studies also showed low smoking-related knowledge and inappropriate attitudes towards SHS exposure among smokers and nonsmokers in China [20–22]. Our findings also shows that parents are aware of the importance of restricting children's exposure to SHS but take inappropriate strategies to prevent SHS at home or in the car, such as opening a window or a door, smoking in a separate room, and using air purifier. These findings are in agreement with Phillips et al. [23] and underscore the need for educational intervention engaging parents and household members with strategies to implement a home and car smoking ban [24]. Preventing children's SHS exposure in public places has always been a difficult task, especially where smoking is not prohibited. The measures taken by some parents (i.e., walking away from smoke) should be encouraged and more awareness raising campaigns to discourage public place smoking should be initiated. Posting no-smoking signs and announcing moderate penalties for smokers caught in public venues was effective in Hong Kong [25], and similar measures should be considered for mainland China.

Some of the parents in our study expressed their frustration about not receiving enough smoking cessation or SHS related information from the pediatricians. While these sentiments reflect the clearly expressed hope among parents of pediatric patients to engage with pediatricians about tobacco use and SHS exposure, they also highlight a large gap in the existing pediatric healthcare delivery system. However it is encouraging to note that a few parents experienced brief advice to quit smoking or reduce the child's SHS exposure even in the absence of any mandatory or systematic requirement for inquiry and recording of parental tobacco use or SHS exposure status of children. Earlier studies reported that lack of knowledge about tobacco control measures and lack of skills and confidence in providing counseling to quit or to reduce SHS exposure were associated with not engaging in tobacco control efforts among physicians in Hong Kong [26] and China [27]. Organized training of pediatricians to build their capacity to provide smoking cessation and SHS exposure reduction counseling to parents, which could be incorporated within the CME programs of the Chinese Pediatric Society/Chinese Academy of Medicine (pediatric chapter), would engage more pediatricians in the SHS exposure reduction effort. A policy strategy at the hospital level to adopt mandatory recording of parental smoking and SHS exposure status within the electronic medical record system could prompt pediatricians to initiate child's SHS exposure reduction conversation with the parents or caregivers [28].

Strengths of our study were not only the diverse range of respondents in terms of age, socioeconomic group, and location (four different cities) but also inclusion of both men and women, smokers and nonsmokers to gather varying views. One limitation was that all participants were recruited from the pediatric departments of Chinese hospitals, limiting the generalizability of the findings to attendees in other departments. However, there is no reason to believe that the views about protecting child's health gathered from parents of young children who attended the pediatric departments would be different from the parents who attend other departments within the hospital.

## 5. Conclusion

The findings of this qualitative study among parents of Chinese children indicate that children's exposure to SHS in the home is shaped by a range of sociocultural influences, gaps in knowledge, attitudes towards SHS, and parental smoking behaviors. The findings also highlight the demand for pediatricians to address parental tobacco use and SHS exposure of children. While there is a need for a nationwide survey to better understand actual SHS exposure reduction practices in the pediatric setting throughout China, the current local findings suggest the need for pediatrician engagement to enhance parental smoking cessation and SHS exposure reduction support for children attending pediatric departments. Enhanced pediatrician training and hospital system change might prompt more pediatricians to engage in SHS exposure reduction conversation with parents of children [29].

## 6. What Is New?

This study assessed nature of antismoking discussions Chinese parents had with their child's pediatricians and how pediatricians might engage with parents in an effort to reduce children's exposure to SHS, supporting the creation of model interventions for developing countries.

## Figures and Tables

**Table 1:** Demographic characteristics of focus group discussion (FGD) participants (*n* = 33).

Characteristics	Focus group discussions (6 FGDs; *n* = 33)	
	Smokers (*n* = 18)	Nonsmokers (*n* = 15)
Gender		
Males	18	4
Females	0	11
Age (mean ± SD)	38.56 ± 16.26	32.93 ± 11.14
Education		
Middle school or below	11	12
High school or above	7	3

**Table 2:** Demographic characteristics of focus groups (*n* = 6).

Group	Participants per group	Gender		Average age	Smoking status			Any family smoker	
		Female	Male		Previous	Current	Never	Yes	No
1	7	5	2	45		2	5	7	
2	5	2	3	32		3	2	5	
3	6	1	5	32	1	3	2	6	
4	5	0	5	49	1	4	1	4	1
5	5	1	4	29		2	3	5	
6	5	2	3	27		3	2	5	

**Table 3:** Typical statements made by parents by key themes.

Attitude towards smoking in front of your children	Attitude towards smoking in the car	Attitude towards smoking and quitting smoking	Knowledge of smoking and SHS	Measures taken to reduce children's exposure to SHS	Experience with and views about pediatricians inquiry about smoking and children's SHS exposure
Senior adults (e.g., grandpa) smoke at home; I cannot say anything to stop them. It is too rude. That's not respectful of senior (male smoker and female nonsmoker).	If spending much time in the car, my friends definitely would smoke; I think it is ok (male smoker).	I tried to quit smoking for many times. Once I stopped smoking for half month, then I went to a dinner with friends. They shared cigarettes to me and I had to accept to show politeness. Also after drinking, it's comfortable to have a smoke (male smoker).	Before my son got sick, I did not know smoking was harmful, and I liked to amuse him with cigarettes and allowed him to try in few occasions (male smoker).	When relatives and friends come to visit and smoke at home, I always tell kids to play outside (male smoker).	If the pediatrician told me some smoking-related health hazards, I would recognize (female nonsmoker).

When friends or relatives come and have a visit, they will smoke. It is unavoidable (male smoker). My hubby's friends always smoke when they drink at home, then the house will be a noisy, smoky bar full of foul air. I am very angry with such mess, but have nothing to do with that (female nonsmoker).	We do not smoke. It's uncomfortable when seeing somebody smoking around our child. That's very annoying (female nonsmoker).	Only aggressive measures can be taken in stopping smoking. My sister's husband is a teacher; his school prohibits smoking by fines and other strict measures. There is no way for him to smoke out of school frequently, so he had to quit smoking (female nonsmoker).	Warning labels in cigarette packet says, “smoking is harmful to health”; but I have no idea what exact harms does smoking cause (male smoker).	Children should be taken away; keep them from exposure to smoking environment (female nonsmoker).	As a smoker, I am accustomed to smell of cigarettes. But when pediatricians smell it, they often told me to quit smoking. But frankly speaking, I did not take it seriously (male smoker).

I smoke even when children at home. When craving come up, I am unable to control, no choice (male smoker).	If children are in the car, I do not let people smoke in it. Without kids, it does not matter (male nonsmoker).	My dad is in his seventies. When I requested him to quit, he said you better ask me to quit eating rather than quit smoking (male nonsmoker).	I would cough when I smell the smoke, I know smoking is harmful but do not know it's so serious (male nonsmoker).	I hide in the bathroom, den, or balcony to smoke (male smoker).	Engagement of pediatricians would be acceptable to parents in reducing child's SHS exposure.

It is a very difficult thing to stop people smoking in front of kids (male smoker and female nonsmoker).	When I drive alone, I definitely smoke. Taking someone else in car, if friends smoke, I will follow (male smoker).	When I was pregnant, my husband quitted smoking in his own accord. Now, he smokes again, and I ask him not to smoke; he said baby was born and smoking did not matter (female nonsmoker).	I am not sure if smoking is really harmful. Why some smokers live longer than nonsmokers? (male smoker).	I try not to smoke and remind others for not smoking in the presence of children (male smoker).	I hope we can get more information about smoking and assistance in quitting smoking at the hospitals (male smoker).

It's get me very upset that my husband smokes at home. I confined him in balcony or bathroom to smoke, but it's still smoky in house (female nonsmoker). I really hate the smoky smell of my father-in-law; it's so strong and really horrible. After smoking, he just goes to hold the baby without mouthwash and I feel bad (female nonsmoker).	When I drive, even kids in the car, I can't help smoking. I just open the window (male smoker).	I know smoking is harmful, but I'm addicted to it (male smoker). When I smoke, I always tell myself this is the last one, but, no, that's just another one (male smoker). In my circle, every men smoke. It's so common. That's life, nothing is serious. I never thought about quitting (male smoker).			
